# The CXCR4/SDF-1 Axis in the Development of Facial Expression and Non-somitic Neck Muscles

**DOI:** 10.3389/fcell.2020.615264

**Published:** 2020-12-22

**Authors:** Imadeldin Yahya, Gabriela Morosan-Puopolo, Beate Brand-Saberi

**Affiliations:** ^1^Department of Anatomy and Molecular Embryology, Ruhr University Bochum, Bochum, Germany; ^2^Department of Anatomy, Faculty of Veterinary Medicine, University of Khartoum, Khartoum, Sudan

**Keywords:** CXCR4, SDF-1, facial expression muscles, non-somitic neck muscles, cell migration

## Abstract

Trunk and head muscles originate from distinct embryonic regions: while the trunk muscles derive from the paraxial mesoderm that becomes segmented into somites, the majority of head muscles develops from the unsegmented cranial paraxial mesoderm. Differences in the molecular control of trunk versus head and neck muscles have been discovered about 25 years ago; interestingly, differences in satellite cell subpopulations were also described more recently. Specifically, the satellite cells of the facial expression muscles share properties with heart muscle. In adult vertebrates, neck muscles span the transition zone between head and trunk. Mastication and facial expression muscles derive from the mesodermal progenitor cells that are located in the first and second branchial arches, respectively. The cucullaris muscle (non-somitic neck muscle) originates from the posterior-most branchial arches. Like other subclasses within the chemokines and chemokine receptors, CXCR4 and SDF-1 play essential roles in the migration of cells within a number of various tissues during development. CXCR4 as receptor together with its ligand SDF-1 have mainly been described to regulate the migration of the trunk muscle progenitor cells. This review first underlines our recent understanding of the development of the facial expression (second arch-derived) muscles, focusing on new insights into the migration event and how this embryonic process is different from the development of mastication (first arch-derived) muscles. Other muscles associated with the head, such as non-somitic neck muscles derived from muscle progenitor cells located in the posterior branchial arches, are also in the focus of this review. Implications on human muscle dystrophies affecting the muscles of face and neck are also discussed.

## Introduction

Remarkably, skeletal muscles in the trunk and head regions differ in a number of important aspects (Lescroart et al., 2010). The primary function of trunk skeletal muscles is locomotion, whereas craniofacial skeletal muscles do not serve in locomotion (Schubert et al., 2019). Instead, they are essential for controlling eye movements, facial expression and mastication (Sambasivan et al., 2011; Schubert et al., 2019). Trunk and head muscles also have distinct embryonic origins (Noden and Francis-West, 2006; Lescroart et al., 2010). Differences can already be observed in the myogenic programmes controlling head and trunk myogenesis (Grifone and Kelly, 2007; Sambasivan et al., 2011; Schubert et al., 2019; Vyas et al., 2020). In addition to these distinguishing criteria, it should also be mentioned that the head mesoderm includes a common progenitor pool contributing to the heart and the skeletal muscles (Vyas et al., 2020). The most remarkable feature of the head musculature is that its connective tissue originates from a different source than that of the trunk muscle (Christ et al., 1982; Noden and Trainor, 2005; Noden and Francis-West, 2006; Masyuk and Brand-Saberi, 2015). Neck muscles link the head to the trunk and derive from dual mesodermal (somitic and non-somitic) origins (Theis et al., 2010; Lescroart et al., 2015; Heude et al., 2018). Interestingly, non-somitic neck muscle share a common set of gene regulatory networks with head muscles and cardiac progenitors of the second heart field (Lescroart et al., 2015). In the trunk, the dorsal domain of the somite, the dermomyotome, retains its epithelial structure for longer and contributes to all the skeletal muscles of the trunk and limbs (Buckingham and Rigby, 2014; Buckingham and Relaix, 2015). The premyogenic progenitor cells delaminate from the dermomyotome (ventrolateral lip) and undertake a long-range migration from the somite to more distant sites of myogenesis such as the limbs, diaphragm and tongue (Vasyutina et al., 2005; Buckingham and Relaix, 2015; Masyuk and Brand-Saberi, 2015). Migration of skeletal muscle progenitor cells is a complex process and involves chemokines and chemokine receptor signaling that allow the cells to stay motile and find their final destination. CXCR4/SDF-1 axis has previously been shown to play a role in the development of migrating muscle progenitor cells of the limb, tongue, pectoral girdle and cloaca (Odemis et al., 2005; Vasyutina et al., 2005; Yusuf et al., 2006; Rehimi et al., 2010; Masyuk et al., 2014). Furthermore, SDF-1 positively regulates the expression of irregular connective tissue markers during limb development and controls the formation of blood vessels in the somite (Abduelmula et al., 2016; Nassari et al., 2017). Recently, we reported that CXCR4/SDF-1 axis has a crucial role in facial and neck muscle development (Yahya et al., 2020b).

In this Review, we intend to provide an update of the research data concerning the origin and gene regulatory networks of vertebrate facial and neck muscles, taking into account the evidence for common embryonic origins of these muscles with heart muscle. We also discuss the roles of SDF-1 and its receptor CXCR4 in the development of skeletal muscles. Since search data concerning the role of chemokines in trunk muscles development have been previously reviewed by Masyuk and Brand-Saberi (2015), we discuss them only briefly here and focus instead on the role of the CXCR4/SDF-1 axis during head muscles development and summarize recent findings of its role in the migration of the second arch-derived and non-somitic neck muscle progenitor cells.

## Head Muscle Origin

**FIGURE 1:**
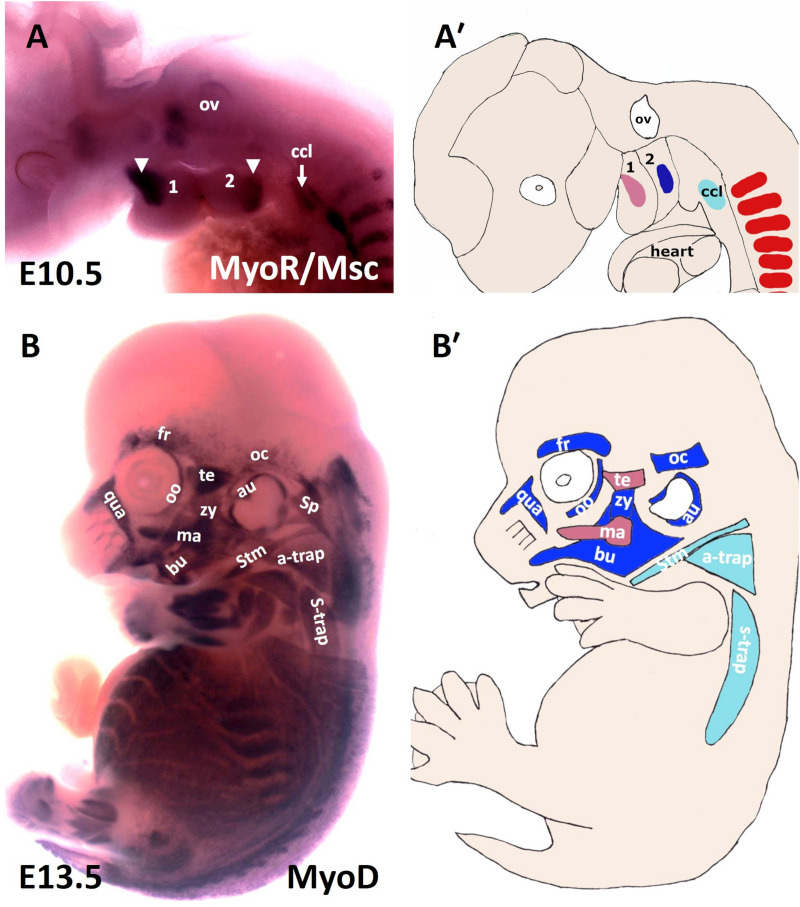
Muscles of the mouse head and neck. **(A)** Lateral view of a stage E10.5 mouse embryo hybridized with a MSC (MyoR) probe. **(A′)** Schematic representation of E10.5 mouse embryo in the panel **(A)**. In pink: first arch-derived muscle anlage. In blue: second arch-derived muscle anlage. In pale blue: cucullaris muscle anlage. MSC marked the myogenic core of the BA1 and BA2. Cucullaris muscle anlage is also labeled with MSC. **(B)** Lateral view of a stage E13.5 mouse embryo hybridized with a MyoD probe. **(B′)** Schematic representation of E13.5 mouse embryo in the panel **(B)**. MyoD is expressed in all branchiomeric muscles and cucullaris muscle anlage. a-trap, acromio-trapezius; au, auricularis; BA1-2, branchial arches 1–2; bu, buccinator; ccl, cucullaris anlage; fr, frontalis; ma, masseter; oc, occipitalis; oo, orbicularis oculi; ov, otic vesicle; qua, quadratus labii; sp, splenius; stm, sternocleidomastoideus; s-trap, spino-trapezius; te, temporalis; zy, zygomaticus.

**FIGURE 2:**
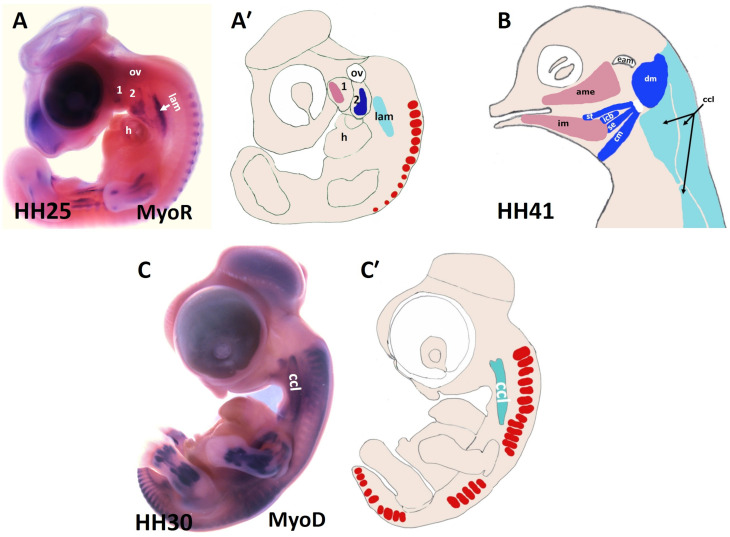
Muscles of the chicken head and neck. **(A,C)** Lateral view of chicken embryos hybridized with DIG-probes for either MyoR **(A)** or MyoD **(C)**. **(A′,C′)** Schematic representation of chicken embryos in the panels **(A,C)**. **(B)** Lateral view of head muscles of a stage HH41 chicken embryo. The MyoD and MyoR delineated the BA1-derived muscles (pink), the BA2-derived muscles (blue) and the cucullaris muscle (pale blue). ame, adductor mandibulae externus; 1-2, branchial arches 1-2; ccl, cucullaris anlage; cm, caudal mylohyoideus; dm, depressor mandibulae; eam, external auditory meatus; h, heart; icb, interceratobranchialis; im, intermandibularis; lam, lateral plate mesoderm; ov, otic vesicle; se, serpihyoideus; st, stylohyoideus.

Trunk and head muscles originate from distinct embryonic regions: trunk muscles from paraxial mesoderm that becomes segmented into somites, but the majority of head muscles are developed from unsegmented cranial mesoderm (Lu et al., 2002; Noden and Francis-West, 2006; Lescroart et al., 2010). Neck muscles span the transition zone between head and trunk (Theis et al., 2010; Lescroart et al., 2015; Sefton et al., 2016; Heude et al., 2018). Even though the myogenic regulatory factors orchestrate a developmental program shared by all body muscles, there is clear evidence that muscle development in the head and trunk differ with regard to the initial phases in myogenic lineage specification (Lu et al., 2002). Craniofacial muscles can be arranged into several groups: (1) muscles that control eye movement (extraocular), (2) muscles in or associated with the head (somite-derived tongue and neck muscles), and (3) branchiomeric muscles that are involved in mastication, facial expression and function of the pharynx and larynx (Lescroart et al., 2010). Branchiomeric muscles originate from the mesodermal core of the branchial arches (BAs), which consists of cells from both cranial paraxial mesoderm (CPM) and lateral splanchnic mesoderm (SPM) (Lescroart et al., 2010). In chicken, CPM cells give rise to the proximal region of the mesodermal core, whereas SPM cells give rise to the mesodermal cells in the distal region of the BAs (Nathan et al., 2008). Mastication and facial expression muscles originate from the mesodermal progenitor cells that are located in the BA1 and BA2 (Figure 1 and Table 1), respectively (Noden and Francis-West, 2006; Lescroart et al., 2015). In avians, the second arch-derived muscles include the muscle of the mandibular depressor, the muscle of columella (stapedial), the constrictor colli, the stylohyoid, the serpihyoid, the mylohyoid (caudal) and the interceratobranchial muscles (Figure 2B and Table 1; McClearn and Noden, 1988). Unlike in mammals, they participate in food uptake by rotating the lower jaw, raising the floor of the mouth, retraction of the hyoid apparatus and intraoral food transport (Jones et al., 2019). Non-branchiomeric head muscles include tongue muscles derived from muscle progenitor cells located in the anterior-most somites, and extraocular muscles derived mainly from the prechordal mesoderm (Kelly et al., 2004; Noden and Francis-West, 2006; Lescroart et al., 2010).

**TABLE 1:** Avian and mouse facial and neck muscle.

		Muscles	References
Avian	First arch	Adductor mandibulae externus Adductor mandibulae caudalis *Pseudotemporalis profundus Pseudotemporalis superficialis* Pterygoideus Protractor of quadrate Intermandibularis (dorsal and ventral)	McClearn and Noden, 1988
	Second arch	Depressor mandibulae Muscle of the columella (stapedial) *Constrictor colli Mylohyoideus caudalis* Serpihyoideus Stylohyoideus Interceratobranchialis	McClearn and Noden, 1988
	Neck	**Cucullaris muscle** (cucullaris capitis and cucullaris cervicis)	Theis et al., 2010
	First arch	Temporalis and Masseter	Lescroart et al., 2010
Mouse	Second arch	Auricularis Buccinator Frontalis, Occipitalis Orbicularis oculi Quadratus labii and Zygomaticus	Lescroart et al., 2010
	Neck (non-somitic)	Acromio-trapezius Spino-trapezius Sternocleidomastoideus	Heude et al., 2018
	Neck (somitic)	**Epaxial neck muscles** (Levator scapulae, Semispinalis, Splenius, suboccipital, postvertebral muscles and rhomboid occipitalis) **Hypaxial neck muscles** (infrahyoid muscles, longus capitis and longus colli)	Heude et al., 2018

## Distinct Origins of the Neck Muscle

There are approximately 80 skeletal muscles in the human neck that control the processes of respiration, vocalization, swallowing and head mobility (Heude et al., 2018). Neck muscles are classified based on their anatomical location within the neck: for instance, cucullaris-derived muscles (Figure 1B), ventral hypaxial muscles, pharyngeal, laryngeal and esophageal striated muscles located medioventrally and epaxial back muscles (Figure 1B and Table 1; Heude et al., 2018). The amniote homolog of the cucullaris muscle is divided dorsoventrally into the larger dorsally positioned (trapezius) muscles and ventrally positioned (sternocleidomastoideus) muscles (Theis et al., 2010). Recently, interest in cervical musculature has significantly increased, with the application of clonal analysis, gene targeting, and molecular profiling studies. The origin of the cucullaris muscle or its mammalian homologs the sternocleidomastoideus and trapezius has been the subject of considerable debate (Theis et al., 2010; Heude et al., 2018). Previous findings in different organisms including mice, lungfish, amphibians and shark suggest that the cucullaris develop from posterior BAs (Matsuoka et al., 2005; Noden and Francis-West, 2006; Ericsson et al., 2013; Diogo et al., 2015; Lescroart et al., 2015; Sefton et al., 2016; Naumann et al., 2017; Noda et al., 2017; Ziermann et al., 2018). In chicken, several embryological origins involving lateral plate mesoderm (Figures 2A,C) and somites have been described for the cucullaris muscles (Huang et al., 2000; Theis et al., 2010; Nagashima et al., 2016). Interestingly, a recent lineage-tracing study in mice using lineage-specific Cre drivers for Pax3, Islet1, Mef2c-AFH (anterior heart field) and Mesp1 suggests that the cucullaris muscle anlage develops as part of the mesodermal core of BA 3-6 and anterior-most somites (S 1–3), but extends caudally into the lateral plate mesoderm and is innervated by the accessory nerve XI (Heude et al., 2018). In their study, the Tajbakhsh group reported that the cucullaris-derived myofibres are not part of the lateral plate mesoderm based on their expression profile (Prx1 lineage). The lateral plate mesoderm instead gives rise to the associated connective tissue of the cucullaris-derived muscles (Heude et al., 2018). Thus, sternocleidomastoideus and trapezius, although being called “non-somitic,” they are of mixed origin, head and somitic mesoderm contribute to their formation.

## Skeletal Muscle Elements of the Head and Neck

**FIGURE 3:**
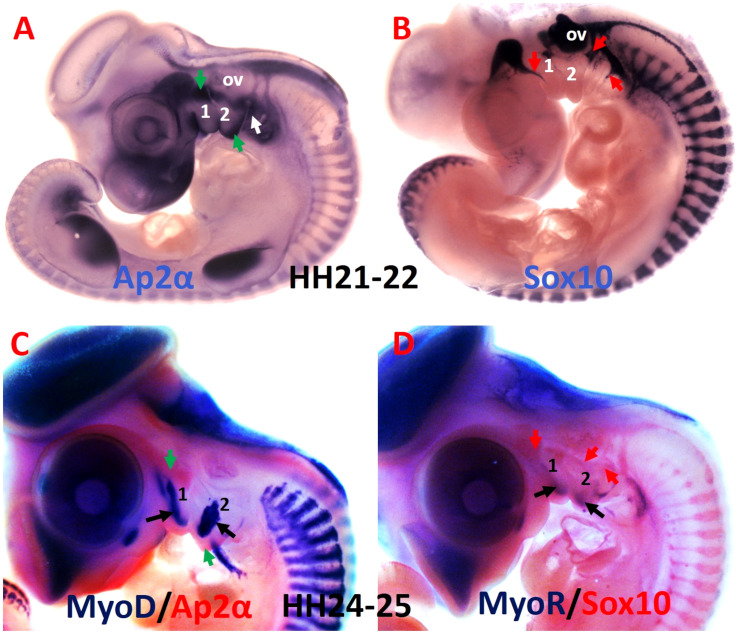
Comparative analysis of the mesodermal and neural crest cell makers. **(A,B)** Whole-mount chicken embryos were hybridized with DIG-labeled probes for Ap2α and Sox10. **(C)** Whole-mount embryos are labeled with MyoD probes in blue and Ap2α probe in red. **(D)** Whole-mount embryos are labeled with MyoR probes in blue and Sox10 probe in red. Ap2α marked the ectomesenchymal neural crest cells (green arrows), whereas Sox10 labeled non-ectomesenchymal neural crest cells (red arrows). The myogenic cells are marked by MyoD and MyoR (black arrows). 1-2, branchial arches 1-2; ov, otic vesicle.

In the body, the central functions of connective tissues are to link up cells and tissues and to support organs. Connective tissue generates a broad range of derivatives, which can be classified into three groups: specialized connective tissue (corresponds to cartilage and bone), loose connective tissue and dense connective tissue, which is subdivided into regular (refers to tendon and ligament) and irregular. Irregular connective tissue includes cartilage perichondrium, muscle epimysium and connective tissue inside the muscle (Clemente, 1985; Omelyanenko et al., 2014; Nassari et al., 2017). BAs are composed of two mesenchymal cell populations (Figure 3), originating from cranial paraxial mesoderm and from the neural crest cells (Grenier et al., 2009). The neural crest cells can be grouped into two categories, ectomesenchymal (Figures 3A,C), and non-ectomesenchymal (Figures 3B,D). The ectomesenchymal neural crest cells migrate into the BAs and form connective tissue, whereas the non-ectomesenchymal crest cells give rise to neurons, glia and pigment cells (Blentic et al., 2008). More recently, however, it has been reported that the neural crest cells give rise to the muscle connective tissue that connect the head and shoulders, while mesodermal cells contribute to attachment sites of muscles linking the trunk and limbs (Heude et al., 2018). Mesodermal and neural crest cells seem to differ in the manner of developing bones: mesodermal cells form endochondral skeleton in the trunk whereas neural crest cells form dermal and endochondral bones in the head (Matsuoka et al., 2005). In zebrafish, Kague et al. (2012) demonstrated neural crest cells contribution to many bones of the craniofacial skeleton and for some later developing cartilage elements, as well as to a subset of myocardial cells. Unlike in other model vertebrates, the trunk neural crest cells in zebrafish have realized their capacity to differentiate into osteoblasts (Kague et al., 2012). In chicken and mouse, neural crest cells are an established source for the vertebrate craniofacial skeleton. Although many similarities between mouse and chicken, there are also clear variations in the contribution of the neural crest cells (Kague et al., 2012). In the chicken, neural crest cells do not contribute to any part of the shoulder girdle (Epperlein et al., 2012). In mouse, neural crest cells give rise to the attachment points of the cleidohyoideus and trapezius muscles inside the shoulder girdle endoskeleton (Matsuoka et al., 2005). In contrast to this finding, a recent study in mouse reported that the neural crest cells reveal restricted contribution to cucullaris attachment sites and do not give rise to osteoblasts at the posterior attachment regions (Heude et al., 2018). This view suggests that the gradient of mesodermal and neural crest cells contributions to neck connective tissue relies on the cellular origin of associated skeletal components (Heude et al., 2018).

## The Genetic Requirements for the Formation of Head and Neck Muscles

**FIGURE 4:**
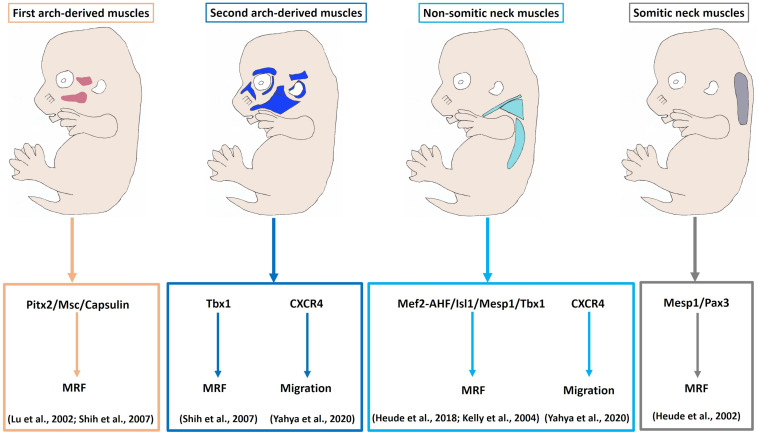
Model of myogenic programs in the facial and neck muscles. Pitx2, Capsulin and Msc are required for the Myf5 and MyoD activation in the BA1-derived muscle progenitor cells. Tbx1 is required for initiation of myogenic progression in the BA2. Non-somitic neck (cucullaris) muscle development is controlled by Mef2c-AHF, Isl1 and Mesp1. The genetic network for somite-derived (epaxial) neck muscles involves both Mesp1 and Pax3 genes. CXCR4 is required for the migration of the BA2-derived and non-somitic neck muscle progenitor cells. MRF, Myogenic regulatory factors.

Myogenic programmes are distinct not only between head and trunk muscles, but also amongst neck muscles. Likewise, within the head muscles, extraocular muscles differ from branchial muscles, and myogenic programmes that lead to the formation of branchial muscles vary considerably among the different BAs (Nathan et al., 2008). A comparison of the molecular signature in the satellite cells of mastication and extraocular muscles to limb muscles in the adult reveals more differences (Sambasivan et al., 2009). Sambasivan et al. reported that EOM are absent in Myf5/Mrf4 double mutants, which shows a unique genetic programm, different from all other skeletal muscles in the embryo. In contrast, BA1-derived muscles are not affected by the inactivation of these two genes. In the limb, Pax3 compensated for the lack of Myf5 and Mrf4 functions (Sambasivan et al., 2009). In their work from Harel et al. (2009) show that the bone morphogenetic protein 4 (BMP4) effectively downregulated myogenic differentiation markers (MyHC and MyoG) in trunk-derived, but less so in head derived satellite cells. Moreover, BMP4 induced greater expression of cardiac markers (Tbx20 and Isl1) in head satellite cells, but not trunk satellite cells. Thus, head satellite cells may retain cardiogenic competence and could provide a future source of cell-based therapy to repair cardiac damage (Rios and Marcelle, 2009). Furthermore, studies of satellite cells from the head and limb muscles exhibit differing regenerative capacities in their response to injury (Pavlath et al., 1998). This lineage heterogeneity is found within the craniofacial muscle progenitor and satellite cells, as a result of their distinct embryonic origins (Harel et al., 2009). Recently, many studies of developmental myogenesis have focused on head and trunk muscles, however, little is known regarding the development of neck muscles. New genetic studies are starting to shed light on the mechanisms governing skeletal myogenesis in the neck (Figure 4). Apart from differences of neck muscle formation based on origin, it has recently been shown that they develop according to unique genetic programmes. A number of recent findings have established that an unexpected diversity occurs among different neck muscle groups (Heude et al., 2018). Such molecular diversity in terms of the expression of specific upstream regulators of the neck myogenic program has begun to be understood in the last few years.

Pax transcription factor family 3 (Pax3) marks trunk skeletal muscle progenitor cells and controls their entry into the muscle differentiation program (Buckingham and Relaix, 2007). In Pax3/Myf5 double mutants trunk muscles are absent, whereas head muscles are not affected (Tajbakhsh et al., 1997). In the head muscle satellite/progenitor cells, the role of Pax3 is replaced by Tbx1, Isl1, Pitx2, Capsulin and MyoR (Shih et al., 2007; Sambasivan et al., 2009, 2011; Theis et al., 2010; Moncaut et al., 2012; Buckingham and Relaix, 2015), indicating that myogenesis in the head is regulated by a distinct mechanism (Sambasivan et al., 2011; Lescroart et al., 2015). Previous studies in chicken, mouse, frog and zebrafish reported that head skeletal muscle satellite cells do not have an earlier history of Pax3 and instead, Pax7 develops later and marks satellite cells (Buckingham and Rigby, 2014; Nogueira et al., 2015). Pax7 mutant (whole body mutant) offspring are viable within three weeks after birth (Mansouri et al., 1996). In Pax7 mutants, skeletal muscle forms normally, whereas facial skeletal structures are affected which could be linked to neural crest cells defect (Mansouri et al., 1996). Pax7 is detected in mandibular adductor (CPM-derived), but not intermandibular muscle (SPM-derived) myoblasts, while Isl1 is detected in the intermandibular muscle (Nathan et al., 2008). In SPM-derived branchiomeric muscle progenitor cells, Isl1 was reported to delay MyHC expression in a manner similar to its expression in undifferentiated second heart field cells (Nathan et al., 2008). Thus, Isl1 might have a role in the control of self-renewal of satellite cells in branchiomeric muscles (similar to the role of Pax7 in trunk) (Nathan et al., 2008). In chicken and turtles, the cucullaris muscle develops in a Pax3-independent manner (Theis et al., 2010). Pax7 is not detected during early cucullaris muscle formation, but is slightly found at stage HH28. Likewise, Myf5 expression starts late (HH26) in the cucullaris muscle compared with trunk and head muscles (Theis et al., 2010). The lack of the early Myf5 and Pax7 expression in the cucullaris muscle reveals that it forms during the period of phasing out of the trunk myogenic programme (Theis et al., 2010).

The pituitary homeobox 2 (Pitx2) is expressed in the head mesoderm and BA1-mesodermal core. In Pitx2 mutants, the EOM and BA1-derived muscles were affected, whereas BA2-derived muscles were merely altered (Shih et al., 2007). Thus, Pitx2 seems to be required for the specification of BA1-derived muscles by controlling MyoR, Capsulin and Tbx1 in early stages (Shih et al., 2007). Likewise, in Capsulin/MyoR double mutant mice BA1-derived muscles were absent, whereas BA2-derived muscle were present (Lu et al., 2002). These findings identify Pitx2, Capsulin and MyoR as unique gene regulators for the formation of BA1 muscles (Lu et al., 2002; Shih et al., 2007). The onset of myogenic program in the BA2 mesodermal core is regulated by Tbx1, which controls Myf5 and MyoD expressions (Kelly et al., 2004; Shih et al., 2008). Analyses of Tbx1 mutant embryos revealed that muscles derived from BA2 were absent (Kelly et al., 2004). In contrast, skeletal muscle progenitor cells derived from BA1 were present (Kelly et al., 2004). Thus, Tbx1 is not necessary for migration of cranial mesodermal progenitor cells into the BA1 (Kelly et al., 2004). In chicken, the expression of Tbx1, MyoR, Pitx2, and Capsulin were found to mark the cucullaris muscle anlagen from its earliest stage (HH14) of development (Theis et al., 2010). In mouse, the above-mentioned recent study indicates that transcription factors involved in trunk myogenesis are not important for the development of anterior somites neck muscles in contrast to more posterior somites neck (hypaxial) muscles (Heude et al., 2018). Interestingly, the cucullaris muscle is formed from progenitor cells that have expressed Mesp1, Mef2-AHFc, Tbx1, and Islet1 markers (transcription factors regulating head muscle formation) (Kelly et al., 2004; Theis et al., 2010; Lescroart et al., 2015; Heude et al., 2018). The absence of transcription factors involved in trunk myogenesis and the late expression of Myf5 and Pax7 supported the hypothesis that the cucullaris muscle develops according to a head muscle programme (Theis et al., 2010). Additionally, mesoderm posterior homolog transcription factor 1, Mesp1, is considered as the master regulator of cardiac mesodermal cells that contribute to both the first and the second heart fields (Chan et al., 2013). Mesp1 is a context-dependent transcription factor, combining the signals and the phase of differentiation to promote other mesodermal lineages, namely cardiac, skeletal muscle and hematopoietic (Saga et al., 1996, 2000; Chan et al., 2013). A recent study in mice demonstrates that Mesp1 widely expresses in the cranial mesoderm and anterior somites 1–6, whereas its expression declines in more posterior somites (Heude et al., 2018). Furthermore, all epaxial/hypaxial neck muscles originate from cranial somitic Mesp1 + cells, while trunk/limb muscles deriving from more caudal somitic Pax3 + cells (Heude et al., 2018).

## Clonal Relationship Between Facial Expression, Non-Somitic Derived and Cardiac Muscles

Cardiac and skeletal muscles are both striated. In tune with this issue, another peculiarity of the head mesoderm is its ability to generate craniofacial and cardiac muscles progenitor cells (Sambasivan et al., 2011; Vyas et al., 2020). In the past, the heart muscle was thought to originate from a single source of myocardial progenitor cells. However, an additional source of common mesodermal progenitor cells that contribute to descendants in both types of striated muscle has been discovered (Tirosh-Finkel et al., 2006; Lescroart et al., 2010, 2015; Chan et al., 2016; Vyas et al., 2020). Retrospective clonal analysis experiments had indicated two branchiomeric muscle lineages, both of which also contribute myocardium. The first lineage derives from BA1 mesoderm and contributes to BA1-derived muscles (temporalis and masseter muscles) and myocardium of the right ventricle. The second lineage gives rise to BA2-derived (facial expression) muscles and outflow myocardium (Lescroart et al., 2010). Likewise, clonal analysis study in the mouse revealed that cardiac progenitor cells in pharyngeal mesoderm of the second heart field share a gene regulatory network with non-somitic neck muscles (Lescroart et al., 2015). In the same year, a different group has identified the third lineage of cardio-pharyngeal mesoderm. They have reported that esophageal striated muscles are derived from pharyngeal mesoderm that contributes to head muscles and derivatives of the second heart field (Gopalakrishnan et al., 2015). In the adult, branchiomeric muscles are equipped with quiescent satellite (stem) cells which are marked by Pax7. These satellite cells however are not derived from the trunk mesoderm (Pax3^+^ lineage). Instead, they are derived from the head mesoderm and continue to express the early head muscle markers (Nogueira et al., 2015). In both chicken and mouse models, lineage studies show that Isl1 + lineage of the SPM generates satellite cells of subset of branchiomeric skeletal muscles, whereas Mesp1 + head mesoderm lineage give rise to satellite cells in extraocular and CPM-branchiomeric muscles (Harel et al., 2009). In addition to lineage distinction, a difference regarding the developmental potential was observed between head and trunk satellite cells. *In vitro* experiments revealed a cardiogenic potential of head, but not trunk-derived satellite cells (Harel et al., 2009). The ability of head satellite cells to retain some of the early head mesoderm properties and contribute to heart muscle have important implications in developing specialized muscle stem cells and cardiac cells for therapy (Nogueira et al., 2015).

## Cell Migration

During embryogenesis, there are several incidents where tissue or organ development depends on precise migration of progenitor cells from their respective sites of emergence (Miller et al., 2008). Cell migration plays a fundamental role in development, regeneration and disease. This migration requires chemokine/chemokine receptor signals that allow the cells to stay motile and find their targets (Vasyutina et al., 2005). Chemokines are small chemoattractant cytokines that are classified according to positioning of certain conserved N-terminal cysteine residues (C) (Pawig et al., 2015; Scala, 2015). The cysteine residues can be adjacent (CC-family) or spaced from each other by one or three amino acids (CXC and CX3C families) (Scala, 2015). Chemokine receptors are classified in CR, CCR, CXCR, and CX3CR receptors in relation to nomenclature aligns with their ligands (Pawig et al., 2015; Pozzobon et al., 2016). Chemokine receptors belong to G protein-coupled receptors, which signal via trimeric G proteins (Pawig et al., 2015). The best described chemokine is SDF-1 (Stromal Derived Factor 1, also known as CXCL12), whose functions are mediated by two chemokine receptors (CXCR4 and CXCR7) (Garcia-Andres and Torres, 2010). Although the first known activity of CXCR4 was the regulation of HIV- infection and cancer metastasis (Bleul et al., 1997; Helbig et al., 2003; Doi et al., 2018; Shanmugam et al., 2018; Bianchi and Mezzapelle, 2020), CXCR4 is also an abundantly expressed chemokine receptor throughout embryogenesis (Yusuf et al., 2005; Yusuf et al., 2006). The functions of the CXCR4/SDF-1 axis during embryogenesis include heart ventricular septum formation, gut vascular morphogenesis, sympathetic precursor cell migration, dentate granule cell migration, B lymphocyte migration, sensory neuron clustering, limb neuromuscular development and palatal osteogenesis (Bagri et al., 2002; Odemis et al., 2005; Kasemeier-Kulesa et al., 2010; Escot et al., 2013; Mahadevan et al., 2014; Terheyden-Keighley et al., 2018; Laparidou et al., 2020; Verheijen et al., 2020; Yahya et al., 2020a).

### CXCR4 Signaling Pathways

Upon SDF-1 binding CXCR4 at the N-terminal domain, the receptor undergoes a conformational change, which activates the associated trimeric G protein. Next, the receptor undertakes a second conformational change that induces G protein subunits to dissociate into Gα subunit and Gβγ dimer (Arnolds and Spencer, 2014; Pozzobon et al., 2016). Each subunit can activate a variety of biological responses such as cell migration, proliferation, survival and differentiation. CXCR4-oriented migration is facilitated by several members of the phosphoinositide 3-kinase (PI3-kinase) family, which can be exerted by both Gα and Gβγ subunits (Ward, 2006; Pozzobon et al., 2016). PI3-kinases promote cell migration and gene transcription by the phosphorylation of pro-survival effector AKT (also known as protein kinase B) (Teicher and Fricker, 2010; Pozzobon et al., 2016). Furthermore, Gβγ dimer can trigger phospholipase C (PLC), leading to calcium mobilization, activation of protein kinase C (PKC) and mitogen associated protein kinase (MAPK) (Arnolds and Spencer, 2014; Pozzobon et al., 2016). The CXCR4/SDF-1 signaling cascade can take various routes, but ultimately leads to cell migration, survival, and proliferation (Arnolds and Spencer, 2014).

We and others have identified the CXCR4/SDF-1 axis as major players in cell migration, proliferation and survival during development of limb and cloacal muscles (Odemis et al., 2005; Vasyutina et al., 2005; Yusuf et al., 2005, 2006; Odemis et al., 2007; Rehimi et al., 2010; Masyuk et al., 2014). In contrast to our understanding of the role of CXCR4/SDF-1 axis in the trunk muscles development, less is known about its role in formation of the head and neck muscles. We have recently provided evidence that disruption of CXCR4/SDF-1 signaling also impairs facial and non-somitic neck muscles formation (Yahya et al., 2020b). This review summarizes the main roles of the CXCR4/SDF-1 axis in skeletal muscle development, concerning the special emphasis on the development of the BA2 and non-somitic neck muscles.

### The CXCR4/SDF-1 Axis Participates in Specific Facial and Neck Muscles Development

Development and patterning of the BAs play key roles in craniofacial formation (Kelly et al., 2004). We have previously documented that both CXCR4 and SDF-1 were expressed in the chicken BAs (Yusuf et al., 2005; Rehimi et al., 2008). However, the function of this axis in the BAs was not clear. Later study documented that cardiac neural crest cells migrating toward BA3 and BA4 express CXCR4 and that SDF-1 shows a complementary expression pattern in the ectodermal cells beside their migratory way (Escot et al., 2013). More recently, it has been recognized that absence of CXCR4 signaling results in misrouting of BAs neural crest cells and massive morphological modifications in the mandibular skeleton, cranial sensory ganglia and thymus (Escot et al., 2016). However, their role in the development of facial muscles development remains to be fully clarified.

**FIGURE 5:**
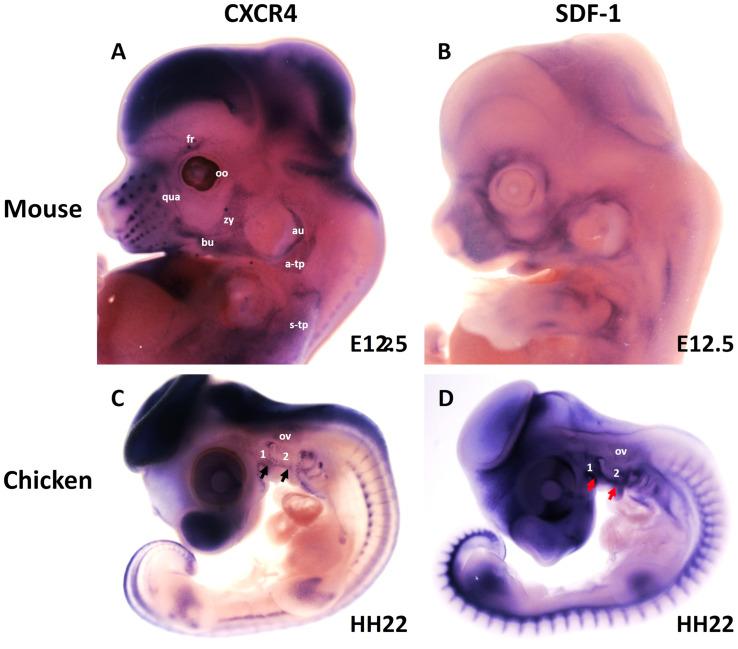
Expression of CXCR4 and its ligand SDF-1 during head and neck muscle development. **(A,B)** Whole-mount *in situ* hybridization of CXCR4 **(A)** and SDF-1 **(B)** in mouse embryos at E12.5. Note labeling of BA2-derived muscles anlage (fr, bu, oo, zy, au, qua) by CXCR4. CXCR4 is also expressed in non-somitic neck muscles (a-trap, s-trap). SDF-1 is expressed in the adjacent mesenchyme of the face. **(C,D)** Whole-mount *in situ* hybridization of CXCR4 **(A)** and SDF-1 **(B)** in chicken embryos at HH22. CXCR4 and SDF-1 are expressed in complementary patterns in the chicken branchial arches. Black and red arrows mark CXCR4 and SDF-1 in the BAs. a-trap, acromio-trapezius; au, auricularis; BA1-2, branchial arches 1–2; bu, buccinator; fr, frontalis; oo, orbicularis oculi; ov, otic vesicle; qua, quadratus labii; s-trap, spino-trapezius; zy, zygomaticus.

**FIGURE 6:**
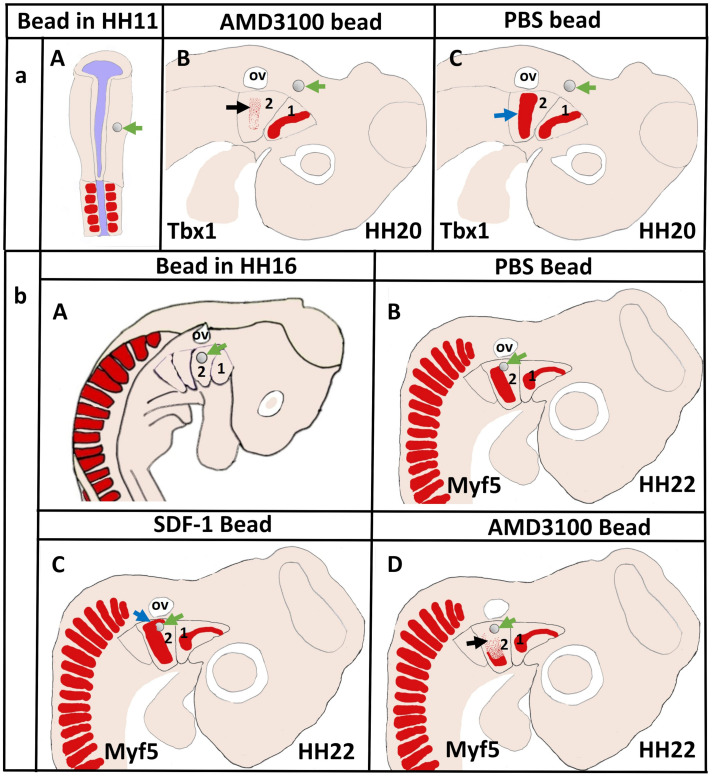
Disruption of CXCR4 signaling in the early head mesoderm and BA2 inhibits migration of the BA2-mesodermal progenitor cells. **(aA)** Schematic representation of chicken embryo at HH11 implanted with beads soaked with the CXCR4 inhibitor AMD3100 or with PBS in head mesoderm. The embryos were re-incubated until they reached stages HH20 and hybridized with a Tbx1 probe. **(aB,aC)** Schematic representation showing the head region and the location of mesodermal cells in the BA2 at HH20. Tbx1 expressing area in the BA2 (black arrow) was diminished in AMD3100-treated embryos **(B)** in comparison with the PBS-treated embryos **(C)**. **(bA)** Schematic representation of embryo at HH16 implanted with beads soaked with the AMD3100 or with SDF-1 in head mesoderm. PBS bead used as control. Myf5−expressing region (blue arrow in **bC**) was increased in SDF-1-treated embryo. The BA2 myogenic core region (black arrow in **bD**) was reduced in AMD3100-treated embryos, but not in PBS-treated embryos **(bB)**. Green arrows show the location of the implanted beads.

**FIGURE 7:**
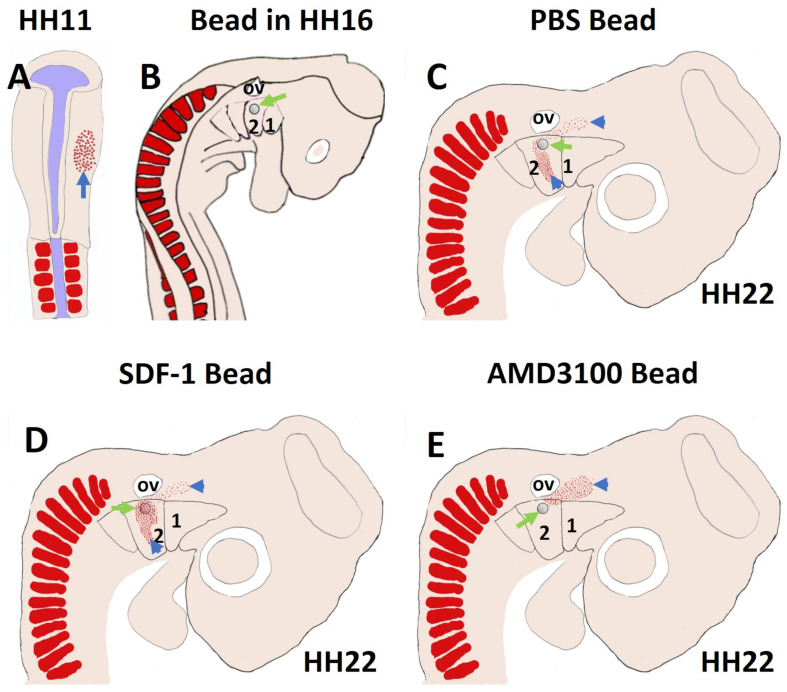
SDF-1 guides the migration of the second arch-derived muscle progenitor cells. To further test the role of CXCR4 in early head muscle development, we injected quail cells into the right side of the head mesoderm of recipient chicken embryos at HH11 **(A)** followed by AMD3100, SDF-1 or PBS beads implantation **(B)**. The quail cells emigrated from the head mesoderm and populated BA2 of hosts. The quail cells were traced by whole-mount immunostaining for QCPN **(C–E)**. Whole-mount immunostaining revealed that the positive quail cells were prevented from entering the BA2 in AMD3100 implanted embryos, but not PBS-treated embryos. In the case of SDF-1 treated embryos, the quail cells were expanded and attracted.

More recently, we revealed that the chemokine receptor CXCR4 and its ligand SDF-1 have a critical role in the facial muscles development in the chicken and mouse embryos. At E10.5, we found that CXCR4 is expressed in the migrating muscle progenitor cells in the core of the BA2 and SDF-1 is detected in the BA2 endoderm in proximity to these progenitor cells (Yahya et al., 2020b). Later, CXCR4 is already transcribed in all BA2-derived muscle progenitor cells, but not BA1-derived muscle progenitor cells (Figure 5A). CXCR4 is also noticed in non-somitic neck muscles (sternocleidomastoideus, s-trapezius and a-trapezius) (Figure 5A). SDF-1 showed a complementary expression pattern in the regions that correspond to the BA2-derived muscle anlagen (Figure 5B) (Yahya et al., 2020b). Thus, these CXCR4 and SDF-1 expression patterns correlate closely with the migration of second arch-derived muscle progenitor cells, as well as the non-somtic neck muscle cells. In mice carrying a mutation in the CXCR4 gene, the BA2-derived muscles were nearly completely missing. Most strikingly, first arch derived muscles were not affected. Interestingly, the cucullaris muscle group (sternocleidomastoideus, s-trapezius and a-trapezius) and part of splenius (non-somitic part) muscles were also impaired (Yahya et al., 2020b). We next turned to the chicken model to check whether CXCR4/SDF-1 axis might be involved in BA2-derived muscle formation. Indeed, CXCR4 is expressed in the BA2 mesodermal core (Figure 5C). CXCR4 positive cells are also observed in the BA1 (Yahya et al., 2020b). AMD3100 is novel antagonists of CXCR4 and it was previously reported to competitively inhibit SDF-1 binding to CXCR4 in various chicken embryo tissues (Katsumoto and Kume, 2011; Mahadevan et al., 2014). Inhibition of CXCR4 by implantation of AMD3100 beads into the cranial paraxial mesoderm (Figure 6aA) disturbed the migration of Tbx1-expressing cells, which then caused a reduction in Tbx1-expressing muscle progenitor cells in the BA2 mesodermal core (Figure 6aB). Moreover, misregulating this signal at a later stage by application of the same inhibitor beads into the BA2, resulted in decreased expression of myogenic markers (Figure 6bD). Conversely, ectopic application of SDF-1 protein (Figure 6bA) (gain-of-function approaches) in the chicken BA2 lead to an attraction of myogenic progenitor cells, which was reflected in an enlargement of the expression domain of myogenic regulatory factors around SDF-1 beads (Figure 6bC; Yahya et al., 2020b). In contrast, control PBS beads didn t show any change in the expression (Figures 6aC,6bB). Additionally, we could show the importance of the SDF-1 for the migration of BA2 cells in chicken embryo by injection of quail cells into the CPM (Figure 7A) at HH11 followed by SDF-1 bead implantation (Figure 7B) at HH16 (Yahya et al., 2020b, c). Application of these SDF-1 beads in the BA2 enhanced the migration of the QCPN-positive cells from the CPM into BA2 (Figure 7D), which led to their accumulation around the SDF-1 source. In contrast, the AMD3100 bead prevented the quail cells from entering the BA2 (Figure 7E). PBS beads did not show any change in the expression (Figure 7C).

It is now well established that the control of myogenesis of BA1 and BA2-derived muscles is differentially regulated (Moncaut et al., 2012). Tbx1 is expressed in the mesodermal core of the BAs, which gives rise to facial expression and non-somitic neck muscles (Kelly et al., 2004). In Tbx1 mutant, the skeletal muscle progenitor cells in the BA2 and caudal BAs are severely disturbed or absent, suggesting that the majority of facial expression and non-somitic neck muscles do not form in Tbx1 null embryos (Kelly et al., 2004). A significant Tbx1 binding site was documented at the CXCR4 promoter (Escot et al., 2016). It has been reported that CXCR4 and SDF-1 are genetically downstream of Tbx1 in the course of BAs colonization by neural crest cells (Escot et al., 2016). Furthermore, CXCR4 and SDF-1 expression levels are reduced in Tbx1 knockout embryos (Escot et al., 2016). At the SDF-1 locus, Pitx2 binding sites were found, but not at the CXCR4 locus (Mahadevan et al., 2014). Additionally, Pitx2 was needed to initiate expression of premyogenic markers in the BA1. In Pitx2 knockout mice embryos, the BA1 muscle precursor cells failed to expand after E9.5, while the BA2 cells were not affected (Shih et al., 2007). Since Tbx1 but not Pitx2 is required for the initiation of the myogenic program in the BA2, we suggest a direct link between CXCR4 and Tbx1 during development of the specific head and neck muscles. Disrupting of this link could ultimately cause many of the facial and neck myopathies.

### The Role of the CXCR4/SDF-1 Axis in the Limb and Cloacal Muscles Development

CXCR4 transcripts were expressed in the somites and lateral plate mesoderm at stages HH9–HH10 (Yusuf et al., 2005). The earliest expression of SDF-1 in the paraxial mesoderm and lateral plate mesoderm was noticed at stage HH12 (Yusuf et al., 2005). Later, CXCR4 was expressed in migrating muscle progenitor cells toward limb buds, whereas SDF-1 was detected in the limb buds mesenchyme (Vasyutina et al., 2005; Yusuf et al., 2006). CXCR4 expression pattern in the migrating limb muscle progenitor cells was restricted to the dorsal and ventral locations (Vasyutina et al., 2005; Yusuf et al., 2005). SDF-1 transcripts are detected in the central limb mesenchyme close to the locations occupied by muscle progenitor cells (Vasyutina et al., 2005). Thus, SDF-1 is needed to sustain CXCR4- expressing skeletal muscle precursors at dorsal and ventral locations (Garcia-Andres and Torres, 2010). In CXCR4 mutant embryos, changes in the distribution and survival of muscle progenitor cells in the dorsal limb were observed (Vasyutina et al., 2005).

We have also reported that SDF-1 is expressed in the cloacal cleft and the proximal region of the hindlimb, while migrating cloacal muscle progenitor cells express CXCR4 (Rehimi et al., 2010). Disruption of CXCR4/SDF-1 signaling in the proximal hindlimb altered their ability to migrate toward the cloaca region and ultimately leads to defects in cloacal muscle development (Rehimi et al., 2010). Moreover, misregulation of CXCR4/SDF-1 signaling impairs dorsal root ganglion neurons and spinal cord motoneurons formation, leading to reduce innervation of the developing mouse limbs. In developing limbs, SDF-1 impact on perichondrium and epimysium involves CXCR4 and vessels. Those new function of CXCR4/SDF-1 axis in the connective tissue and neuromuscular development might open new perspectives to a better understanding of the fibrosis mechanisms and neuromuscular disorders in the trunk region.

### Role of CXCR4/SDF-1 Axis in Muscle Regeneration and Diseases

Alongside CXCR4/SDF-1 axis influences on muscle development, the impact of this axis on muscle regeneration and diseases has been under closer investigation (Brzoska et al., 2006, 2012, 2015; Perez et al., 2009; Bobadilla et al., 2014; Kowalski et al., 2017; Kasprzycka et al., 2019). Many studies indicated that muscle committed stem/progenitor and satellite cells express CXCR4 and their migration depends on an SDF-1gradient (Ratajczak et al., 2003; Kucia et al., 2006; Odemis et al., 2007; Brzoska et al., 2012). Human cord blood stem cells mobilization following transplantation depends on SDF-1 overexpression in damaged skeletal muscle (Brzoska et al., 2006). Analysis of endogenous and *ex vivo* cultured satellite cells revealed that SDF-1 stimulated mobilization of the myoblasts in CXCR4-dependent manners (Brzoska et al., 2012). Furthermore, a role for the CXCR4/SDF-1 axis in muscle maintenance and repair has recently been discovered (Bobadilla et al., 2014). CXCR4 and SDF-1 are highly expressed in damaged muscles. Delayed muscle regeneration in injured muscle treated with CXCR4 inhibitor was observed, whereas the application of SDF-1 protein accelerated repair (Bobadilla et al., 2014). Recent study by Brzoska et al. (2015) suggested that SDF-1 improves skeletal muscle regeneration by increasing expression of the tetraspanin CD9 adhesion protein involved in myoblasts fusion (Brzoska et al., 2015). A more recent study by Kowalski et al. (2017) showed that SDF-1 altered the actin organization via Ras-Related C3 Botulinum Toxin Substrate 1 (Rac-1), cell division control protein 42 (Cdc42), and focal adhesion kinase (FAK). They also indicated that SDF-1 altered the transcription profile of genes encoding the most potent regeneration factors involved in cells movement and adhesion (Kowalski et al., 2017). Additionally, expression of CXCR4 in engrafted myogenic progenitors derived from the adult skeletal muscle into dystrophic fibers was up-regulated, suggesting its involvement in the cell-based therapy (Perez et al., 2009).

Facio-scapulo-humeral muscular dystrophy (FSHD), an autosomal dominant disease, affects in its early stage the muscle of the eye (orbicularis oculi muscle) and the mouth (orbicularis oris muscle). Later, the weakness of the muscles progresses to the upper torso, the muscles connecting shoulder girdle to the thorax, in particular the trapezius muscle. Being the third most common muscular dystrophy with an incidence of 12:100.000 (Deenen et al., 2014) after Duchenne muscular dystrophy and myotonic dystrophy, it will be very interesting to find out if the disruption of the CXCR4/SDF-1 axis as essential regulators of BA2-derived and non-somitic neck muscle development, is responsible for this illness. Although at first view, the affected muscle groups seem to be unrelated, a common origin of the facial and neck muscles was recently demonstrated. The critical role of muscle development and repair by the CXCR4/SDF-1 axis suggests that it may be a promising therapeutic target for particular muscular dystrophies (Hunger et al., 2012).

## Conclusion

It has been more than 2 decades since the emergence of a new concept of the cardiopharyngeal field, which proposes that the pharyngeal mesoderm gives rise to second heart field (SHF) and branchiomeric muscles. In recent years, a wide range of investigations have been carried out to reveal the relationship between branchiomeric and heart muscles. However, the mechanisms of the migration of SHF progenitor cells has been largely ignored. We have recently revealed the importance of the CXCR4/SDF-1 for the migration of myogenic progenitor cells during development of branchiomeric muscles. It will be of great interest to investigate the role of the CXCR4/SDF-1 axis in migration of the newly discovered SHF progenitor cells. Understanding the mechanisms that control SHF cells migration is certainly crucial if these cells are intended to be used for therapeutic applications.

## Author Contributions

IY prepared the figures. All authors listed have contributed to the article and approved the submitted version.

## Conflict of Interest

The authors declare that the research was conducted in the absence of any commercial or financial relationships that could be construed as a potential conflict of interest.
